# Diagnostic efficacy of remnant cholesterol inflammatory index in diabetic kidney disease: machine learning approaches

**DOI:** 10.3389/fnut.2025.1642358

**Published:** 2025-11-25

**Authors:** Xili Xie, Xueyu Li, Haifeng Li, Yan Gao, Feng Zhao, Chen Jia

**Affiliations:** Department of Disease Prevention and Control, General Hospital of Northern Theater Command, Shenyang, Liaoning, China

**Keywords:** diabetic kidney disease, remnant cholesterol, inflammatory, machine learning, diagnostic model

## Abstract

**Background:**

Emerging evidence indicates that remnant cholesterol (RC) and inflammation play a crucial role in diabetic kidney disease (DKD) pathogenesis. The association and diagnostic efficacy of remnant cholesterol inflammatory index (RCII), integrating RC and inflammatory markers, with DKD remains underexplored.

**Methods:**

This cross-sectional study analyzed data from the National Health and Nutrition Examination Survey (NHANES) 2015–2020, including 5,943 participants. DKD was defined by diabetes, urine albumin to creatinine ratio (ACR) ≥ 30 mg/g and an estimated glomerular filtration rate (eGFR) < 60 mL/min/1.73 m^2^. RC was calculated as total cholesterol minus high-density and low-density lipoprotein cholesterol, while RCII was derived by multiplying RC by high-sensitivity C-reactive protein (hs-CRP). Logistic regression and restricted cubic spline analysis were used to evaluate associations and dose–response relationship between RC and RCII and DKD. We assessed RCII diagnostic efficacy measured by five machine learning algorithms.

**Results:**

Our study observed 1,014 cases of DKD (17.06%), with a higher prevalence among males (14.1%) compared to females (11.7%). The highest RC (OR: 2.73, 95% CI: 2.12–3.52, *P* for trend<0.001) and RCII (OR: 2.29, 95% CI: 1.77–2.97, *P* for trend <0.001) levels were significantly associated with increased DKD risk after full adjustment. The result showed both overall and nonlinear positive correlations between the risk of DKD and both RC (*P* for overall <0.001, *P* for nonlinear = 0.049) and RCII (*P* for overall <0.001, *P* for nonlinear <0.001). Machine learning models incorporating RCII and traditional risk factors demonstrated robust diagnostic efficacy, with extreme gradient boosting (XGBoost) achieving the highest AUC values in the testing set (AUC: 0.953).

**Conclusion:**

Our study suggested RCII was a novel and promising biomarker for DKD risk. Its integration into diagnostic models may enhance early identification and personalized prevention strategies for DKD, addressing a critical need in diabetes management.

## 1 Introduction

Diabetic kidney disease (DKD) is a major and growing complication of diabetes, marked by a gradual deterioration in kidney function that frequently results in end-stage renal disease (ESRD) (1). As the global prevalence of diabetes continues to rise, so does the burden of DKD, posing serious challenges to healthcare systems worldwide (2, 3). The association between metabolic dysregulation associated with diabetes and the subsequent development of renal impairment has drawn considerable attention in both clinical and research settings (4, 5). Early identification and risk stratification of DKD are critical for implementing timely interventions to slow disease progression and improve outcomes. Consequently, it is crucial to discover new biomarkers that can improve early diagnosis and risk assessment, ultimately guiding more personalized and effective prevention strategies.

Remnant cholesterol (RC) has gained attention as an important contributor to the pathogenesis of cardiovascular diseases (CVD) and metabolic disorders (6, 7). RC represents the cholesterol content of triglyceride-rich lipoproteins, including very-low-density lipoproteins (VLDL) and chylomicron remnants (8), which are known to contribute to atherosclerosis (9). There is a growing body of evidence that RC is not only a risk factor for CVD but also influences the development of diabetic complications, such as cardiovascular outcomes in individuals with diabetes and DKD (10, 11). Additionally, inflammation is a well-established driver of DKD pathogenesis, contributing to glomerular injury, tubulointerstitial fibrosis, and progressive renal function decline (12). Given the interplay between lipid metabolism and inflammation in the development of diabetic complications, there is growing interest in composite biomarkers that integrate these two pathways (13, 14). The remnant cholesterol inflammatory index (RCII) was a novel biomarker combining RC levels with inflammatory markers (15). By capturing both lipid abnormalities and inflammatory activity, RCII may offer deeper insights into the pathophysiological processes underlying DKD and improve risk stratification beyond traditional biomarkers (16). However, despite its promising theoretical foundation, research on the association between RCII and DKD is notably lacking. To date, no studies have systematically investigated the diagnostic efficacy of RCII for DKD, leaving a significant gap in our understanding of its clinical utility.

To address this gap, our study investigated the association of RC and RCII with DKD in a nationally representative data in the USA, and the dose–response relationship of RC and RCII on DKD. We also explored the diagnostic efficacy of a combination of traditional potential risk factors and RCII by using machine learning algorithms. By addressing these objectives, our study aims to provide robust evidence on the role of RCII as a novel biomarker for DKD, offering a potential tool for DKD diagnostic and targeted prevention strategies in DKD populations.

## 2 Materials and methods

### 2.1 Study population

The National Health and Nutrition Examination Survey (NHANES) is a comprehensive program designed to assess the health and nutritional status of non-institutionalized individuals in the United States. The research used publicly accessible NHANES data, applying a cross-sectional design with a complex, multistage, stratified sampling method to guarantee the U.S. population’s representativeness. Initially, 25,531 participants were enrolled across two consecutive cycles, 2015–2016 and 2017–2020, which were included in the analysis. Following the application of exclusion criteria, the study population was finalized by excluding those under 20 years of age (*n* = 10,580) and pregnant women (*n* = 157). Furthermore, participants with incomplete data on estimated glomerular filtration rate (eGFR), albumin to creatinine ratio (ACR), albumin (ALB), serum creatinine (Scr), diabetes status, or essential laboratory parameters such as high-sensitivity C-reactive protein (hs-CRP), total cholesterol (TC), high-density lipoprotein cholesterol (HDL-C), and low-density lipoprotein cholesterol (LDL-C) were also excluded from the analysis. The final analysis comprised 5,943 participants (Supplementary Figure S1). This carefully selected cohort enabled a rigorous investigation of the relationships between remnant cholesterol, inflammatory markers, and diabetic kidney disease. The study protocol was approved by the NCHS Ethics Review Board, and all participants provided written informed consent. The study abided by the Declaration of Helsinki principles.

### 2.2 Assessment of RC and RCII

RC was calculated as the difference between TC and the sum of HDL-C and LDL-C, using the formula (17):

$$\mathrm{RC}\;(\mathrm{mg}/\mathrm{dL})=\mathrm{TC}\;(\mathrm{mg}/\mathrm{dL})-(\begin{array}{l} \mathrm{HDL}-C\;[\mathrm{mg}/\mathrm{dL}]\\ +\mathrm{LDL}-C\;[\mathrm{mg}/\mathrm{dL}] \end{array})$$

This calculation was performed using laboratory data obtained from the NHANES database.

RCII was created by combining RC levels with hs-CRP, a recognized indicator of systemic inflammation. RCII was calculated as (15):

RCII=RC(mg/dL)×hs−CRP(mg/L)/10

Both RC and RCII were treated as continuous variables in the analysis. To facilitate clinical interpretation, RC and RCII were also categorized into quartiles based on their distribution in the study population (15).

### 2.3 Outcome ascertainment: DKD

DKD was diagnosed by confirming both diabetes mellitus and kidney dysfunction. Diabetes mellitus was determined following the American Diabetes Association (ADA) guidelines (18), which included: a self-reported diagnosis by a healthcare provider, current use of antidiabetic medications, or hemoglobin A1c (HbA1c) levels of 6.5% or higher.

Diagnostic criteria for DKD: (1) Confirmed diagnosis of diabetes, and (2) urine albumin to creatinine ratio (ACR) ≥ 30 mg/g or estimated glomerular filtration rate (eGFR) ≤ 60 mL/min/1.73 m^2^, or both (19). Kidney dysfunction was evaluated using the eGFR, which was derived from the Chronic Kidney Disease Epidemiology Collaboration (CKD-EPI) formula (20).

$$\begin{array}{l} \text{eGFR}=141\times \min(\frac{\mathrm{Scr}}{\kappa },1)\times \max{\left(\frac{\mathrm{Scr}}{\kappa },1\right)}^{-0.129}\\ \times 0.993^{\mathrm{Age}}\times 1.018(\text{if female})\times 1.159(\text{if black}) \end{array}$$

In the equation, Scr represents serum creatinine levels (mg/dL), with *κ* values set at 0.9 for males and 0.7 for females. The term ‘min’ refers to the smaller value between Scr/*κ* and 1, while ‘max’ denotes the larger value between Scr/κ and 1.

An eGFR < 60 mL/min/1.73 m^2^ was considered indicative of impaired renal function. Participants were classified as having DKD if they met the criteria for diabetes mellitus and had either reduced eGFR (< 60 mL/min/1.73 m^2^).

### 2.4 Covariates

Sociodemographic information, such as age (years), gender (male or female), race (Mexican American, Other Hispanic, Non-Hispanic White People, Non-Hispanic Black, or Other), marital status (married or other), education level (≤high school diploma or >high school diploma), family poverty income ratio (PIR) (<1 or ≥1), smoking status (yes or no), and drinking status (yes or no) were gathered using standardized NHANES questionnaires. Physical measurements included body mass index (BMI), systolic blood pressure (SBP), and diastolic blood pressure (DBP). Hypertension was classified based on either a self-reported diagnosis by a healthcare provider or blood pressure readings meeting the International Society of Hypertension criteria (SBP ≥ 140 mmHg and/or DBP ≥ 90 mmHg) (21). Diabetes duration (years) is defined as the age at which the doctor diagnosed diabetes minus the age at which the individual was enrolled in the study.

### 2.5 Statistical analysis

All analyses were conducted using appropriate NHANES sampling weights, strata, and clustering variables, following established statistical protocols. To impute missing covariate data, the ‘na.roughfix’ function within the ‘randomForest’ package was utilized. To assess the robustness of our findings regarding missing data, we performed a sensitivity analysis using multiple imputation by chained equations (MICE) with 5 imputed datasets and pooled results according to Rubin’s rules. Categorical data were expressed as counts (N) and proportions (%), and comparisons were made using the chi-square test. In continuous variables, means were expressed with standard deviations (SD) with *t*-test, or medians were expressed with interquartile ranges (IQR) with Mann–Whitney’s U test.

We assessed the association of RC and RCII of DKD by using logistic regression analysis. As a reference group, the lowest quartile was used in all analysis. Model 1 was adjusted for none. Age and gender were controlled for in Model 2. Model 3 additionally considered race, educational level, marital status, PIR, smoking status, drinking status, BMI, hypertension, and diabetes duration. The results were expressed as odds ratio (OR) and 95% confidence intervals. *P* for trends were also established and the associations between per 1-SD RC or RCII increase and odds of DKD were also examined. Interaction and stratified analyses were conducted according to covariates. To examine the dose–response relationship between RC, RCII, and DKD, restricted cubic spline (RCS) models were applied. Sensitivity analyses were conducted to ensure the reliability of the findings. We also explored the associations of RC and RCII with HbA1c, ACR, and eGFR by using multiple linear regression.

The dataset was split into training and testing subsets in a 7:3 ratio. Multivariable logistic regression was used to identify traditional clinical features independently associated with DKD in the training set. The candidate variables included in this screening process were age, gender, race, educational level, marital status, PIR, smoking status, drinking status, hypertension, diabetes duration, and BMI. Variables with a significance level of *p* < 0.05 in the multivariable analysis were retained as significant traditional risk factors for subsequent model construction. Five machine learning methods, including logistic regression, random forest, k-nearest neighbors (KNN), extreme gradient boosting (XGBoost), and light gradient boosting machine (LightGBM), were utilized to construct diagnostic models that included both traditional risk factors and RCII. The best-performing model, as determined by the highest Area Under the Curve (AUC) in the testing set, was further interpreted using Shapley Additive Explanations (SHAP) to elucidate the direction and magnitude of each feature’s contribution to the model’s predictions. To further assess the generalizability of our optimal model, we performed an external validation as a sensitivity analysis. The best-performing model, trained on the NHANES dataset, was applied without retraining to an independent external dataset derived from the 2011 wave of the China Health and Retirement Longitudinal Study (CHARLS).

Statistical analysis was conducted utilizing R 4.4.2.

## 3 Results

### 3.1 Characteristics of participants

The characteristics of 5,943 participants stratified by DKD status are presented in Table 1. The study identified 1,014 cases of DKD (17.06%). Participants with DKD were significantly older (62.58 ± 11.93 vs. 48.33 ± 17.23, *p* < 0.001) and had higher BMI (32.43 ± 7.74 vs. 29.26 ± 7.23, *p* < 0.001). Notable racial differences were observed (*p* = 0.001), with Mexican American showing the highest DKD prevalence (22.37%). The DKD group exhibited worse metabolic profiles, including HbA1c (7.54 ± 1.78 vs. 5.54 ± 0.62, *p* < 0.001), and lower eGFR (57.38 ± 10.36 ± 7.59, *p* < 0.001). Additionally, DKD participants had significantly higher hs-CRP, RC and RCII levels (*p* < 0.001). Additionally, Supplementary Table S1 also showed characteristics of study participants before imputation.

**Table 1:** Characteristics of study participants according to diabetic kidney disease.

Characteristics	Overall	Non-DKD	DKD	*p*
*N* (%)	5,943	4,929 (82.94)	1,014 (17.06)	
Age, years, mean ± SD	50.77 ± 17.30	48.33 ± 17.23	62.58 ± 11.93	**<0.001** ^***^
Gender				**<0.001** ^***^
Male	2,889 (48.61)	2,332 (80.72)	557 (19.28)	
Female	3,054 (51.39)	2,597 (85.04)	457 (14.96)	
Race				**0.001** ^**^
Mexican American	845 (14.22)	656 (77.63)	189 (22.37)	
Other Hispanic	702 (11.81)	554 (78.92)	148 (21.08)	
Non-Hispanic White People	2011 (33.84)	1711 (85.08)	300 (14.92)	
Non-Hispanic Black	1,383 (23.27)	1,178 (85.18)	205 (14.82)	
Other	1,002 (16.86)	830 (82.83)	172 (17.17)	
Educational level				**<0.001** ^***^
≤High school diploma	2,618 (44.05)	2075 (79.26)	543 (20.74)	
>High school diploma	3,325 (55.95)	2,854 (85.83)	471 (14.17)	
Marital status				**0.001** ^**^
Married	3,350 (56.37)	2,729 (81.46)	621 (18.54)	
Other	2,593 (43.63)	2,200 (84.84)	393 (15.16)	
PIR				0.603
<1	1,120 (18.85)	923 (82.41)	197 (17.59)	
≥1	4,823 (81.15)	4,006 (83.06)	817 (16.94)	
Smoking status, *N* (%)				**0.001** ^**^
Yes	2,573 (43.29)	2086 (81.07)	487 (18.93)	
No	3,370 (56.71)	2,843 (84.36)	527 (15.64)	
Drinking status, *N* (%)				**<0.001** ^***^
Yes	4,482 (75.42)	3,854 (85.99)	628 (14.01)	
No	1,461 (24.58)	1,075 (73.58)	386 (26.42)	
Hypertension, *N* (%)				**<0.001** ^***^
Yes	3,235 (54.43)	1932 (71.34)	776 (28.66)	
No	2,708 (45.57)	2,997 (92.64)	238 (7.36)	
Diabetes duration, years, mean ± SD	10.66 ± 5.47	10.19 ± 3.69	12.92 ± 10.16	**0.002** ^**^
HbA1c, %, mean ± SD	5.88 ± 1.19	5.54 ± 0.62	7.54 ± 1.78	**<0.001** ^***^
ACR, mg/g, mean ± SD	53.94 ± 358.03	26.26 ± 163.34	188.50 ± 774.77	**<0.001** ^***^
ALB, g/L, mean ± SD	56.34 ± 346.96	31.12 ± 208.81	178.94 ± 689.84	**<0.001** ^***^
Scr, mg/dL, mean ± SD	132.17 ± 81.88	133.47 ± 84.11	125.82 ± 69.71	**0.007** ^**^
eGFR, mL/min/1.73 m^2^, mean ± SD	56.29 ± 10.22	57.38 ± 10.36	51.00 ± 7.59	**<0.001** ^***^
BMI, kg/m^2^, mean ± SD	29.80 ± 7.39	29.26 ± 7.23	32.43 ± 7.74	**0.013** ^*^
TC, mg/dl, mean ± SD	185.85 ± 40.81	188.17 ± 39.71	174.56 ± 44.08	**0.001** ^**^
HDL-C, mg/dL, mean ± SD	54.41 ± 16.50	56.0 ± 0.6	50.4 ± 0.9	**<0.001** ^***^
LDL-C, mg/dL, mean ± SD	110.17 ± 35.84	113.1 ± 0.9	103.1 ± 2.0	**<0.001** ^***^
hs-CRP, mg/l, median (IQR)	4.21 ± 8.32	3.82 ± 7.38	6.12 ± 11.72	**<0.001** ^***^
RC, mg/dL, median (IQR)	21.26 ± 12.71	20.29 ± 12.34	25.99 ± 13.42	**<0.001** ^***^
RCII, median (IQR)	9.26 ± 18.98	7.88 ± 15.53	15.97 ± 29.74	**<0.001** ^***^

Data are presented as number (%), mean ± standard deviation (SD), or median (interquartile range). DKD, diabetic kidney disease; BMI, body mass index; HbA1c, glycated hemoglobin; ACR, albumin to creatinine ratio; ALB, albumin; Scr, serum creatinine; eGFR, estimated glomerular filtration rate; TC, total cholesterol; HDL-C, high-density lipoprotein cholesterol; LDL-C, low-density lipoprotein cholesterol, hs-CRP, high-sensitivity C-reactive protein; RC, remnant cholesterol; RCII, remnant cholesterol inflammatory index. *p < 0.05, **p < 0.01, ***p < 0.001. *p*-values which are lower than the statistical significance of 0.05 have been bolded.

### 3.2 Associations of RC and RCII with DKD

**Figure 1:**
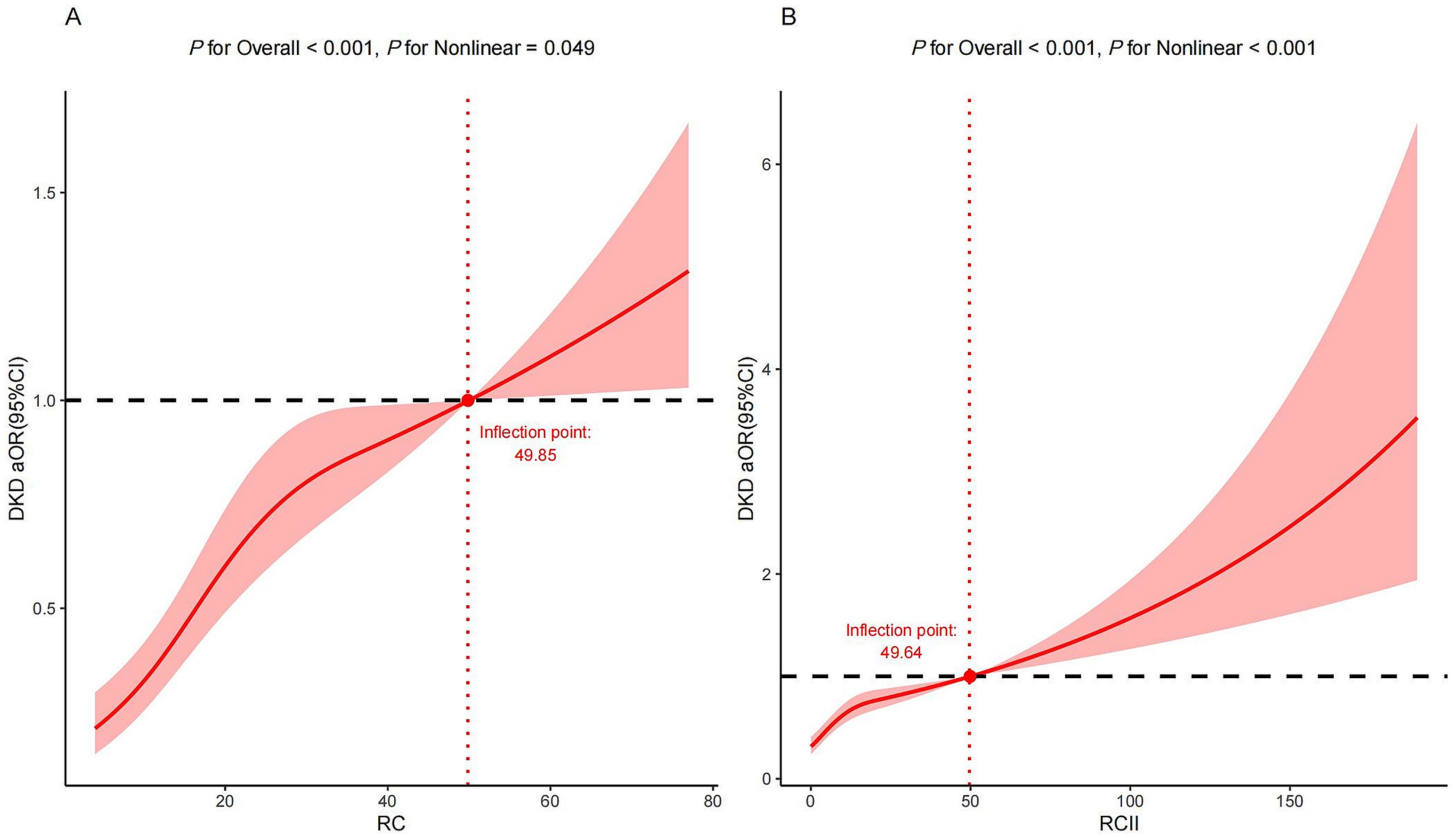
Restricted cubic splines for associations of RC and RCII with the risk of diabetic kidney disease. Restricted cubic spline plots show the association between **(A)** RC and **(B)** RCII with the adjusted odds ratios (solid lines) and 95% confidence intervals (shaded areas) for diabetic kidney disease. The *P* for overall effect tests the global statistical significance of the association (linear or nonlinear), while the *P* for nonlinear tests the specific nonlinear component of the dose–response relationship.

Table 2 presents the associations between RC and RCII levels and DKD risk across three adjusted models. After adjusting for all covariates, elevated RC levels showed a significant positive association with DKD risk (OR: 2.73, 95% CI: 2.12–3.52, *P* for trend <0.001). Additionally, each 1 standard deviation (SD) increase in RC levels was linked to a higher risk of DKD (OR: 1.36, 95% CI: 1.27–1.47). Similarly, higher RCII levels were correlated with increased DKD risk (OR: 2.29, 95% CI: 1.77–2.97, *P* for trend <0.001), and per 1 SD rise in RCII levels correlated with greater DKD risk (OR: 1.32, 95% CI: 1.22, 1.42). Moreover, the spline regression models further validated the presence of both linear and nonlinear positive correlations between the risk of DKD and both RC (*P* for overall <0.001, *P* for nonlinear = 0.049) and RCII (*P* for overall <0.001, *P* for nonlinear <0.001), as illustrated in Figure 1. Notably, inflection points were identified at RC = 49.85 and RCII = 49.64, beyond which the risk of DKD increased markedly. In the supplementary analyses, we further examined the associations of RC and RCII with various metabolic and renal parameters. The analysis of glycemic control indicators revealed strong positive associations of RC and RCII levels with HbA1c (Supplementary Table S2). Higher RC and RCII levels were associated with the increased level of ACR (Supplementary Table S3). Regarding renal function, higher RC and RCII levels were associated with decreased eGFR (Supplementary Table S4). The component analysis of RCII (Supplementary Table S5) indicated that hs-CRP, TC, HDL-C, and LDL-C all contributed to observed associations with DKD risk. The significant associations of RC and RCII with DKD risk remained robust after further adjustment for lipid-lowering and antidiabetic drug use (both *P* for trend < 0.001) in Supplementary Table S6. The sensitivity analysis using multiple imputations showed the RCII and RC were also positively associated with the risk of DKD in Supplementary Table S7.

**Table 2:** Associations of RC and RCII with the risk of diabetic kidney disease.

Characteristics	Model 1	Model 2	Model 3
	OR (95%CI)	OR (95%CI)	OR (95%CI)
RC			
Quartile 1	1.0 (Reference)	1.0 (Reference)	1.0 (Reference)
Quartile 2	**1.93 (1.52, 2.43)**	**1.58 (1.24, 2.02)**	1.41 (1.09, 1.82)
Quartile 3	**3.13 (2.51, 3.91)**	**2.48 (1.96, 3.13)**	**2.02 (1.57, 2.60)**
Quartile 4	**4.14 (3.32, 5.15)**	**3.49 (2.77, 4.39)**	**2.73 (2.12, 3.52)**
*P* for trend	**<0.001**	**<0.001**	**<0.001**
Per-SD	**1.47 (1.39, 1.56)**	**1.47 (1.38, 1.57)**	**1.36 (1.27, 1.47)**
RCII			
Quartile 1	1.0 (Reference)	1.0 (Reference)	1.0 (Reference)
Quartile 2	**1.50 (1.19, 1.89)**	1.23 (0.97, 1.57)	1.06 (0.82, 1.37)
Quartile 3	**2.28 (1.83, 2.83)**	**1.94 (1.54, 2.44)**	1.49 (1.16, 1.91)
Quartile 4	**3.53 (2.86, 4.35)**	**3.47 (2.78, 4.33)**	**2.29 (1.77, 2.97)**
*P* for trend	**<0.001**	**<0.001**	**<0.001**
Per-SD	**1.42 (1.32, 1.52)**	**1.48 (1.37, 1.60)**	**1.32 (1.22, 1.42)**

Model 1: Adjusted for none.

Model 2: Adjusted for age, gender (male; female).

Model 3: Adjusted for age, gender (male; female), race (Mexican American; Other Hispanic; Non-Hispanic Black; Non-Hispanic White People; Other), educational level, (≤High school diploma; >High school diploma), marital status, (Married; Other), PIR (<1; ≥1), smoking status (Yes; No), drinking status (Yes; No), BMI, Hypertension (Yes; No), and diabetes duration.

### 3.3 Stratified analyses

Stratified analyses revealed significant interactions between RC/RCII levels and various demographic and traditional risk factors associated with DKD risk (Table 3). For RC, the strongest associations with DKD were observed among females (OR: 4.61, 95% CI: 3.08–6.90), non-Hispanic Black individuals (OR: 5.64, 95% CI: 3.19–9.97), above high school diploma (OR: 3.32, 95% CI: 2.28–4.82), other marital status (OR: 3.19, 95% CI: 2.12–4.79) and those with hypertension (OR: 2.61, 95% CI: 1.93–3.54). Significant interaction effects were noted for all covariates and RC (*P*-interaction <0.05).

**Table 3:** Stratified analysis of the associations of RC and RCII with the risk of diabetic kidney disease.

Subgroup	RC		RCII	
	Quartile 4 *vs.* Quartile 1	*P*-interaction	Quartile 4 *vs.* Quartile 1	*P*-interaction
Gender		**<0.001**		**<0.001**
Male	**2.00 (1.42, 2.81)**		**1.90 (1.34, 2.94)**	
Female	**4.61 (3.08, 6.90)**		**2.84 (1.90, 4.25)**	
Race		**<0.001**		**0.020**
Mexican American	**2.15 (1.13, 4.08)**		**2.57 (1.30, 5.08)**	
Other Hispanic	1.57 (0.73, 3.37)		1.83 (0.87, 3.88)	
Non-Hispanic White People	**2.67 (1.66, 4.30)**		**1.78 (1.11, 2.83)**	
Non-Hispanic Black	**5.64 (3.19, 9.97)**		**3.99 (2.22, 7.20)**	
Other	1.67 (0.90, 3.09)		1.80 (0.96, 3.38)	
Educational level		**<0.001**		**<0.001**
≤High school diploma	**2.32 (1.64, 3.29)**		**1.79 (1.25, 2.56)**	
>High school diploma	**3.32 (2.28, 4.82)**		**2.96 (2.03, 4.30)**	
Marital status		**<0.001**		**0.006**
Married	**2.44 (1.76, 3.37)**		**2.15 (1.54, 3.00)**	
Other	**3.19 (2.12, 4.79)**		**2.51 (1.66, 3.80)**	
PIR		**<0.001**		**<0.001**
<1	**3.44 (2.36, 5.01)**		**2.65 (1.49, 4.74)**	
≥1	**2.19 (1.54, 3.11)**		**2.77 (2.08, 3.67)**	
Smoking status, *N* (%)		**<0.001**		**<0.001**
Yes	**1.88 (1.30, 2.72)**		**1.88 (1.30, 2.72)**	
No	**2.86 (1.99, 4.12)**		**2.86 (1.99, 4.12)**	
Drinking status, *N* (%)		**<0.001**		**<0.001**
Yes	**2.64 (1.94, 3.60)**		**2.65 (1.91, 3.66)**	
No	**2.95 (1.89, 4.59)**		**1.71 (1.11, 2.64)**	
Hypertension, *N* (%)		**<0.001**		**<0.001**
Yes	**2.61 (1.93, 3.54)**		**2.21 (1.63, 3.01)**	
No	**2.50 (1.57, 3.96)**		**1.97 (1.21, 3.22)**	

Adjusted for age, gender (male; female), race (Mexican American; Other Hispanic; Non-Hispanic Black; Non-Hispanic White; Other), educational level, (≤High school diploma; >High school diploma) marital status (Married; Other), PIR (<1; ≥1), smoking status (Yes; No), drinking status (Yes; No), BMI, Hypertension (Yes; No), and diabetes duration.

For RCII, the strongest associations were observed among females (OR: 2.84, 95% CI: 1.90, 4.25), non-Hispanic Black individuals (OR: 3.99, 95% CI: 2.22–7.20), and those with hypertension (OR: 2.21, 95% CI: 1.63–3.01). Significant interaction effects were also noted for all covariates and RCII (*P*-interaction <0.05).

### 3.4 DKD risk diagnostic model combining traditional risk factors and RCII

**Figure 2:**
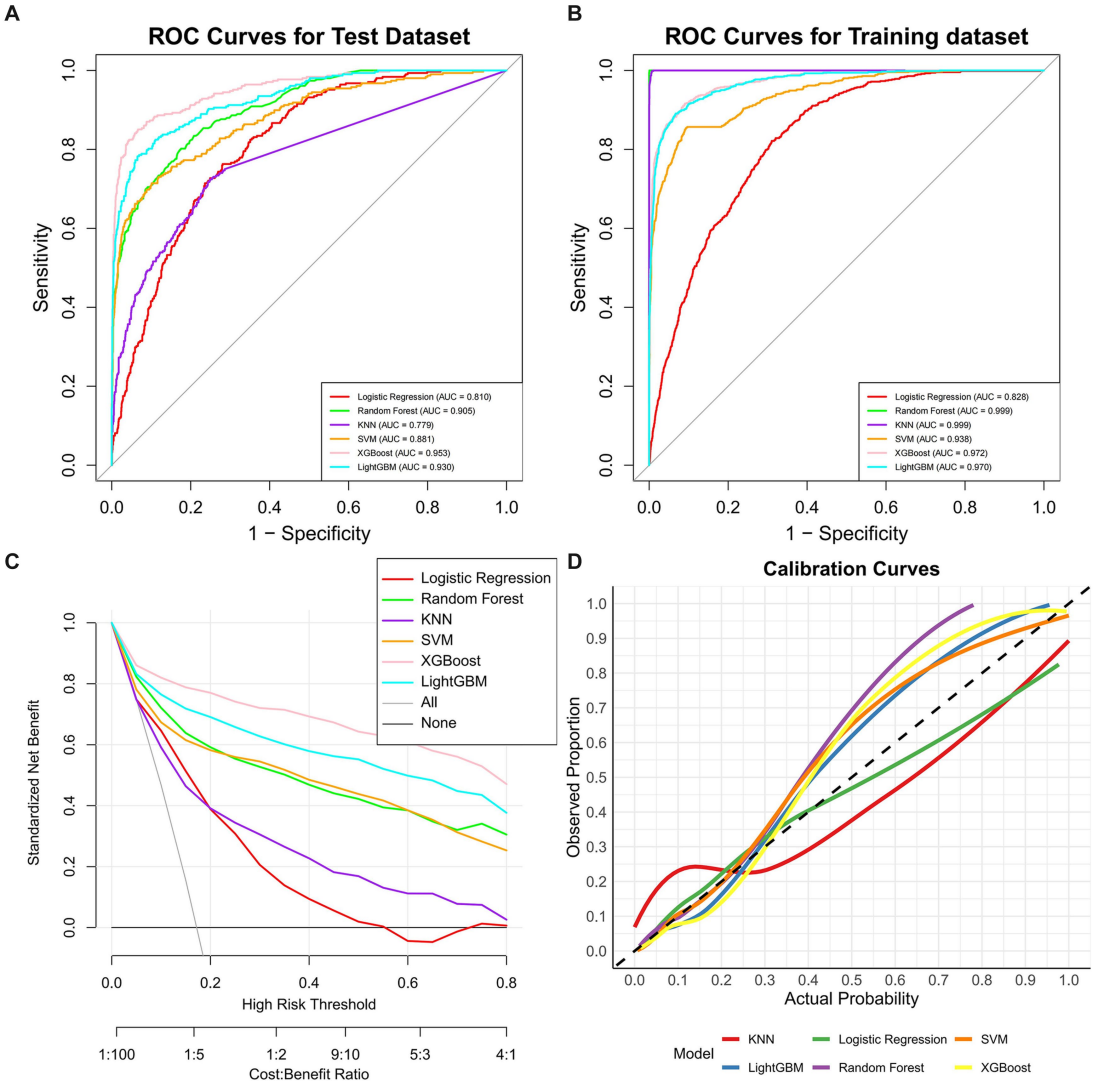
Performance of machine learning (ML) models using RCII and traditional risk factors diagnosing diabetic kidney disease. **(A)** Receiver Operating Characteristic (ROC) curves in the training set; **(B)** ROC curves in the testing set; **(C)** Decision curve analysis; **(D)** Calibration curves.

The multivariate logistic regression analysis (Supplementary Table S9) identified several traditional risk factors significantly associated with DKD, including age, gender, race, educational level, marital status, drinking status, hypertension, diabetes duration, and BMI (*p* < 0.05). These factors, along with RCII, were incorporated into machine learning models to diagnose DKD. The study population was divided into a training set (*n* = 4,161) and a testing set (*n* = 1782) (Supplementary Table S10). Figure 2 illustrates the performance of ML models incorporating RCII and traditional risk factors for DKD. In the training set (Figure 2A), the models demonstrated excellent diagnostic efficacy, with RF (AUC: 0.999), and KNN (AUC: 0.999). XGBoost also performed excellent discrimination (AUC: 0.972), followed by LightGBM (AUC: 0.970), SVM (AUC: 0.938), LR (AUC: 0.828). In the testing set (Figure 2B), XGBoost (AUC: 0.953), LightGBM (AUC: 0.930), RF (AUC: 0.905) showed the highest and most consistent performance. SVM also performed excellent discrimination (AUC: 0.881), followed by LR (AUC: 0.810) and KNN (AUC: 0.778). The calibration curves (Figure 2C) and DCA (Figure 2D) further confirmed the clinical utility of the models, with XGBoost demonstrating robust calibration and net benefit across a range of risk thresholds. These results highlight the effectiveness of combining RCII with traditional risk factors in ML models for DKD diagnoses, with XGBoost emerging as the most reliable approach. Moreover, the accuracy of seven machine learning models in the training and testing sets is summarized in Supplementary Table S11. The sensitivity analysis using multiple imputations showed the XGBoost was still the most reliable approach (Supplementary Table S12; Supplementary Figure S2). In the external validation conducted among the CHARLS participants, the diagnostic performance of all machine learning models was suboptimal (AUCs ranging from 0.513 to 0.594) (Supplementary Figure S3), but the positive association was still observed between RCII and DKD risk (OR: 1.42, 95% CI: 1.23–1.64) (Supplementary Table S8).

**Figure 3:**
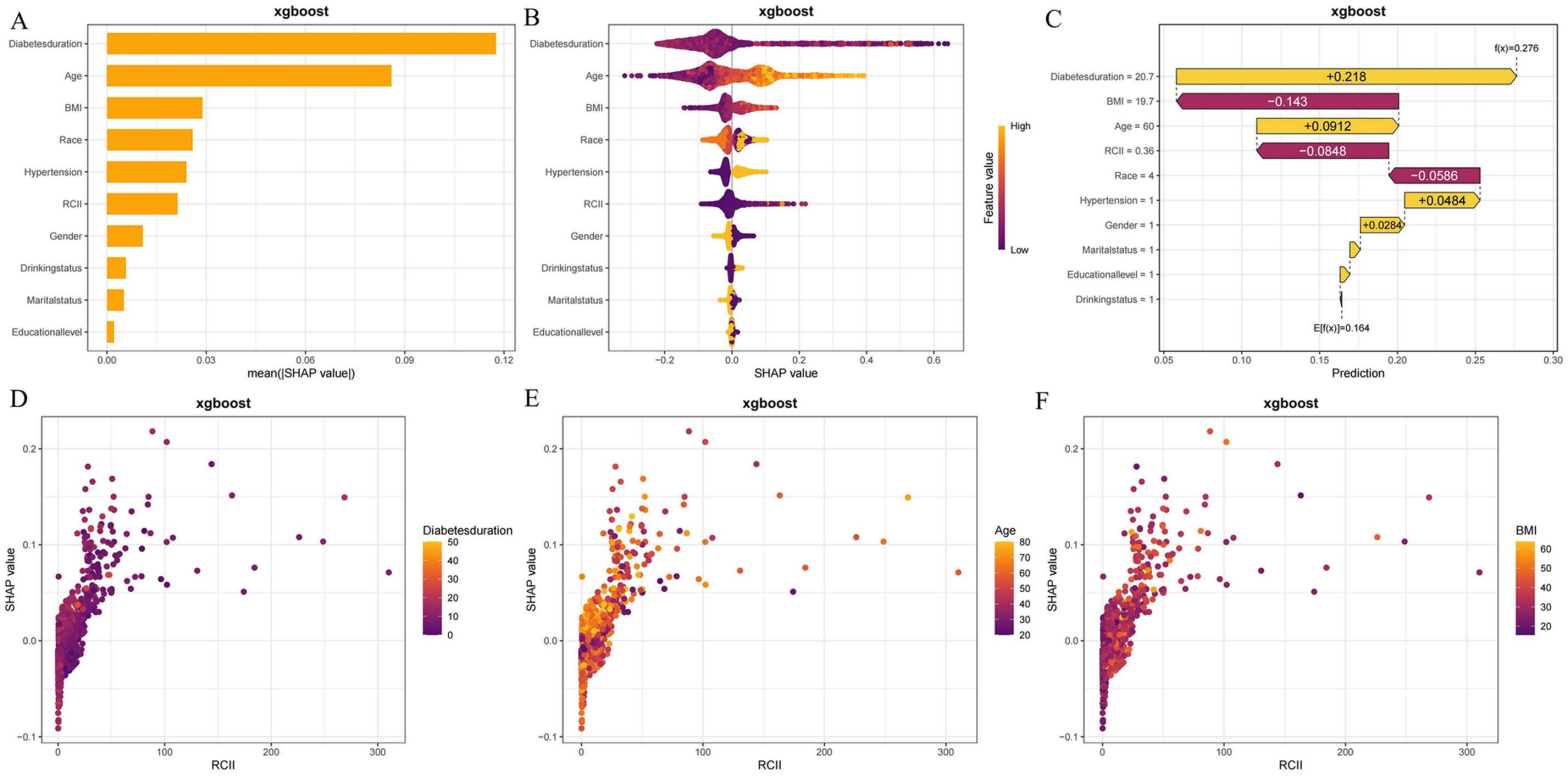
XGBoost explanation by the Shapley Additive Explanation (SHAP) method. **(A)** Order plot of variable importance for SHAP analysis; **(B)** Statistical graph of variable contribution in SHAP analysis; **(C)** Waterfall plot; **(D)** SHAP dependence plots of RCII and diabetes duration; **(E)** SHAP dependence plots of RCII and age; **(F)** SHAP dependence plots of RCII and BMI.

As illustrated in the SHAP summary bar plot (Figure 3A), the relative importance of features in the model was assessed using mean SHAP values, ranked from highest to lowest: diabetes duration, age, BMI, race, hypertension, RCII, gender, drinking status, marital status, educational lever, emerged as the influential features. The SHAP summary dot plot (Figure 3B) further demonstrates the direction and magnitude of each feature’s impact on model diagnose, revealing that variables such as diabetes duration, age, BMI, race, hypertension, RCII significantly elevated the risk of DKD. Additionally, the SHAP waterfall plot (Figure 3C) details the contribution of each feature to the model’s diagnose of DKD for the third participant. The SHAP values in the plot quantitatively demonstrate how each feature influences the diagnostic outcome, with specific values indicating either positive or negative contributions. Notably, a diabetes duration of 20.7 years, an age of 60 years and the presence of hypertension showed significant positive impacts, contributing +0.218, +0.0912, +0.0484 to the diagnosis, respectively. Conversely, a BMI of 19.7 and an RCII level of 0.36 exerted negative effects on the diagnosis, with respective contributions of −0.143 and −0.0848. Figures 3D–F presents a comparative analysis of the top three features against the actual RCII values and their corresponding SHAP values, demonstrating that features with positive SHAP values (>0) are associated with increased diagnostic probabilities in the model, thereby indicating an elevated risk of DKD. The longer diabetes duration, the older individuals, the greater the BMI and the higher the RCII level, the classification was DKD.

## 4 Discussion

This study provides robust evidence supporting the role of remnant cholesterol (RC) and the remnant cholesterol inflammatory index (RCII) as novel biomarkers for diabetic kidney disease (DKD) risk. Our findings demonstrate that elevated RC and RCII levels are significantly associated with an increased risk of DKD, with dose–response relationships. Furthermore, the integration of RCII with traditional risk factors in machine learning models showed strong diagnostic efficacy, underscoring its clinical utility for early identification and risk stratification in DKD populations.

The robust positive association between RC and DKD risk observed in our study corroborates the growing body of evidence establishing lipid abnormalities as critical contributors to diabetic complications. The association was supported by multiple population-based studies across diverse ethnic groups. A Chinese cohort study demonstrated significant associations between RC levels and both DKD and ESRD (22). A FinnDiane study prospectively also observed that the higher level of RC was linked to DKD risk and severe diabetic retinopathy (23). Furthermore, longitudinal data from a Chinese diabetes cohort revealed that both baseline and cumulative RC exposure were positively correlated with DKD development (24). Moreover, the pathological significance of RC extends beyond renal outcomes, as evidenced by its association with increased CVD mortality in participants with type 2 diabetes and established DKD (25). The mechanistic basis for these observational studies lied in RC’s biological properties as a component of triglyceride-rich lipoproteins. A previous study has shown that participants with chronic kidney disease (CKD) have increased production of triglyceride-rich lipoproteins, leading to reduced clearance (26). Moreover, RC is closely linked to renal function progression and the occurrence of cardiovascular events, suggesting that RC may be a new non-invasive marker for predicting ESKD risk (22). Notably, the differential strength of association observed across demographic subgroups - particularly in females, non-Hispanic Black individuals, and hypertensive patients - underscores the importance of population-specific risk stratification in DKD management. These findings indicate that RC significantly contributes to the onset and progression of diabetic nephropathy. Its association with inflammation and cardiovascular risk further underscores the importance of managing RC in diabetic patients (27). These findings offer new perspectives for clinical practice, suggesting the need for further research into the specific mechanisms of RC in diabetic nephropathy and potential therapeutic strategies.

Our study also highlighted the novel RCII biomarker, which integrates RC with systemic inflammation, offers a more comprehensive assessment of DKD risk. Inflammation is a well-recognized driver of DKD progression, contributing to both glomerular damage and tubulointerstitial fibrosis. The development of renal fibrosis is driven by intricate interactions between immune cells and resident kidney cells, which secrete pro-fibrotic cytokines and growth factors, promoting fibrosis. Macrophages, for instance, play a dual role in renal inflammation and fibrosis. They can adopt a pro-inflammatory phenotype that exacerbates renal injury, or an anti-inflammatory phenotype that aids in repair. However, persistent inflammation frequently results in advancing renal fibrosis, potentially leading to end-stage renal disease (28). Additionally, the inflammatory milieu in DKD is influenced by various factors, including metabolic dysfunction and hemodynamic alterations. These elements intensify inflammation and promote fibrosis by triggering processes like epithelial-mesenchymal transition (EMT) and inducing cell cycle arrest in tubular epithelial cells. The activation of pathways like mTOR signaling in macrophages further enhances the inflammatory response and fibrosis-associated EMT, highlighting the intricate link between inflammation and fibrosis in DKD (29). Research underscores the involvement of inflammation in diabetic nephropathy, highlighting how immune cells, cytokines, and chemokines play key roles in initiating and advancing the condition. These inflammatory mediators create a proinflammatory microenvironment that exacerbates kidney damage, leading to increased fibrosis and progression to ESKD (30). Additionally, the combination of lipids and inflammation may be more likely to cause the occurrence of DKD. A study examining the association between remnant cholesterol and CKD highlighted the mediating role of inflammation. It was found that remnant cholesterol and preinflammatory markers had a combined effect on CKD, emphasizing the importance of inflammation in the relationship between remnant cholesterol and renal dysfunction (31). Studies has demonstrated a strong link between glomerular filtration rate, inflammation, and lipid metabolism genes in human diabetic nephropathy, indicating that dysregulated lipid metabolism is a key factor in the advancement of DKD (32). Our results contribute to the expanding evidence highlighting the interaction between lipid metabolism and inflammation in the development of DKD. From a mechanistic perspective, the RCII-DKD association may be explained through several pathways. Elevated RC levels induce oxidative stress and activate transcription factors such as nuclear factor kappa-B (NF-κB), promoting the release of proinflammatory cytokines (e.g., TNF-α, IL-1β, IL-18) and establishing a chronic low-grade inflammatory state (31, 33). Furthermore, high RC levels activate the NLRP3 inflammasome, leading to the maturation and release of inflammatory cytokines such as IL-1β, which exacerbates inflammatory damage in renal tissues (34, 35). The synergistic effect of RC-driven lipid toxicity and subsequent inflammatory activation creates a vicious cycle that promotes endothelial dysfunction, glomerulosclerosis, and tubulointerstitial fibrosis, ultimately accelerating the progression of DKD. Additionally, the RCII may have therapeutic implications, as pharmacological interventions that lower RC levels, such as statins and PCSK9 inhibitors, have been shown to concurrently inhibit NF-κB signaling and attenuate renal inflammation (36). This suggests that targeting RC-related pathways could not only ameliorate dyslipidemia but also directly mitigate the inflammatory drivers of DKD progression, providing a dual therapeutic benefit. The RCII quantitatively integrates these two interconnected processes, providing a comprehensive biomarker that reflects the dual hit of dyslipidemia and inflammation in DKD pathogenesis. By demonstrating a strong association between RCII and DKD risk, our study provides further validation for the role of remnant cholesterol and systemic inflammation as key contributors to renal injury in diabetes. The dose–response relationships observed between RC, RCII, and DKD risk underscore the potential of these biomarkers to improve risk stratification and early detection. Furthermore, the stratified analyses revealed significant interactions between RCII and key demographic variables, with notably stronger associations observed in females and non-Hispanic Black populations. This sexual dimorphism in lipid metabolism is further evidenced by the distinct lifelong patterns of lipid accumulation and exposure in females, characterized by variations during the menstrual cycle, pregnancy, lactation, and the postmenopausal period, which collectively shape a unique cardiovascular risk profile (37). Furthermore, females appear to be disproportionately affected by diabetes, chronic kidney disease, and autoimmune inflammatory conditions, potentially amplifying the detrimental interplay between dyslipidemia, inflammation, and end-organ damage (37). The pronounced association in non-Hispanic Black individuals might reflect the combined impact of socioeconomic disparities, healthcare access limitations, and potentially higher genetic susceptibility to cardiometabolic complications (38–40). These findings collectively underscore the need for tailored strategies in DKD prevention and management that account for these population-specific differences.

Our study not only identified a significant positive correlation between RC and hyperglycemia but also demonstrated a robust positive association between RCII and hyperglycemia, providing direct evidence linking this novel composite biomarker to glucose metabolic disorders. Previous study revealed the relationship between RCII and diabetes is mechanistically grounded in the well-established role of RC as an independent risk factor for type 2 diabetes (T2DM), even after adjustment for traditional lipid parameters (41). Epidemiological studies have consistently demonstrated that elevated RC levels significantly increase T2DM risk, with a 28–48% higher risk per 1 mmol/L increase in RC (42). This association remains robust in individuals achieving conventional lipid targets, where those with high RC (>0.8 mmol/L) face a 4.04-fold higher diabetes risk compared to those with low RC (41). Crucially, chronic inflammation serves as a key mediator in this relationship, with genetic evidence from Mendelian randomization studies confirming that elevated RC directly triggers low-grade inflammation (e.g., increasing C-reactive protein by 28%) and promotes insulin resistance (43, 44). Notably, our findings are supported by established mechanisms linking RC to insulin resistance (IR), a core pathological process in diabetes development. RC levels demonstrate a strong positive correlation with IR severity, as evidenced by significantly higher RC concentrations in moderate-to-severe IR groups compared to mild IR groups (45). Mechanistically, elevated RC contributes to *β*-cell dysfunction through multiple pathways: it directly inhibits insulin secretion, disrupts normal glucose metabolism, and reduces β-cell proliferation capacity (46). These mechanisms collectively establish RC as both a biomarker and functional contributor to diabetes pathogenesis through IR-mediated pathways.

Machine learning approaches have been employed to biomarkers into diagnostic models for DKD. For example, a study utilizing machine learning techniques identified serum uric acid, urea, phosphorous, and other metabolites as significant factors of diabetic kidney disease progression (47). Another research effort created a digital twin model using generalized metabolic fluxes to forecast chronic kidney disease in type 2 diabetes, demonstrating the promise of combining metabolic profiles for improved risk assessment (48). However, to date, no studies have systematically investigated the development of DKD diagnostic models that integrate traditional risk factors with the novel RCII. The unique advantage of RCII lies in its dual-pathophysiological targeting, which distinguishes it from other reported metabolic-inflammatory biomarkers. While previous composite markers often focus on single pathways or general metabolic fluxes (49, 50), RCII directly reflects the synergistic contribution of two key processes in DKD, including lipid-rich remnant particle deposition and chronic inflammatory response. This is supported by cardiovascular research, where the combination of high residual cholesterol and elevated hs-CRP was associated with markedly increased risks of cardiovascular disease (44.9%) and coronary artery disease (57%), along with shorter survival times (51). Furthermore, RC has been shown to outperform traditional lipid markers such as triglycerides and LDL-C in predicting cardiovascular events (AUC 0.919 vs. 0.818 and 0.669, respectively) (52). In diabetic populations, RC correlates positively with inflammatory markers including hs-CRP and fibrinogen, and their combination improves the prediction of short-term cardiovascular outcomes (53). Unlike single-metabolite or general flux-based biomarkers, RCII integrates these two potent risk dimensions into a single interpretable index, offering a more holistic and mechanistically grounded tool for risk stratification. Logistic regression and XGBoost emerged as the most reliable models in our study, with consistent AUC values in both training and testing sets. These findings underscore the potential of combining novel biomarkers like RCII with advanced analytical methods to enhance DKD risk diagnosis. The clinical utility of these models was further supported by calibration curves and decision curve analysis, which confirmed their robustness across a range of risk thresholds. This approach not only improves risk stratification but also provides a framework for personalized prevention strategies, enabling early intervention in high-risk populations.

To our knowledge, this is the first study to comprehensively explore the association between RCII and DKD risk, offering new perspectives on the interplay between lipid metabolism and inflammation in DKD development. It is also the first to create and validate machine learning-based DKD diagnostic models that include RCII alongside conventional risk factors. Using advanced analytical techniques, we showed that models incorporating RCII outperform traditional ones, underscoring their potential as a biomarker for early DKD risk assessment. However, this study has limitations. Its cross-sectional nature limits causal conclusions, and while NHANES data is nationally representative, it may not fully reflect global population diversity. Furthermore, although we attempted external validation using the CHARLS database, the performance was suboptimal, likely attributable to substantial differences in ethnicity, environmental exposures, and age structure between the study populations. This finding underscores the necessity for further validation and investigation across diverse populations in the future. Third, the ‘na.roughfix’ method was used for imputing missing covariate data due to its computational efficiency in the machine learning workflow. Although this method provides a practical single imputation, it does not account for the uncertainty inherent in the imputation process. However, our sensitivity analysis using multiple imputation yielded highly consistent results, which strengthens the confidence in our primary findings. Additionally, single measurements of RC and hs-CRP may not represent long-term exposure. Future longitudinal studies are necessary to confirm these findings and investigate the temporal dynamics between RCII and DKD progression. Lastly, despite adjusting for numerous confounders, residual confounding from unmeasured factors, such as environmental influences, cannot be completely excluded. Furthermore, although we adjusted for lipid-lowering and antidiabetic medications, data on the use of specific anti-inflammatory agents were not available, which represents a potential source of unmeasured confounding.

## 5 Conclusion

RCII represents a promising and novel biomarker for DKD diagnosis, and our study provides robust evidence for a strong, dose-dependent association between RCII levels and the risk of DKD. Its integration into diagnostic models may improve early identification and personalized prevention strategies, addressing a critical need in diabetes management. These results emphasize the need to target both lipid metabolism and inflammation in DKD prevention and demonstrate the value of advanced analytics in enhancing diagnostic models. Additional studies are needed to validate these findings and assess the practical application of RCII across diverse populations.

## Data Availability

Publicly available datasets were analyzed in this study. This data can be found here: https://www.cdc.gov/nchs/nhanes/index.htm.

## Glossary

DKD: Diabetic kidney disease
ESRD: End-stage renal disease
RC: Remnant cholesterol
CVD: Cardiovascular diseases
CHARLS: China Health and Retirement Longitudinal Study
VLDL: Very-low-density lipoproteins
RCII: Remnant cholesterol inflammatory index
MICE: Multiple imputation by chained equations
NHANES: National Health and Nutrition Examination Survey
eGFR: Estimated glomerular filtration rate
hs-CRP: High-sensitivity C-reactive protein
TC: total cholesterol
HDL-C: High-density lipoprotein cholesterol
LDL-C: Low-density lipoprotein cholesterol
ADA: American Diabetes Association
HbA1c: hemoglobin A1c
FBG: Fasting blood glucose
CKD-EPI: Chronic Kidney Disease Epidemiology Collaboration
PIR: Poverty income ratio
BMI: Body mass index
SBP: Systolic blood pressure
DBP: diastolic blood pressure
SD: Standard deviations
IQR: interquartile ranges
RCS: Restricted cubic spline
KNN: K-nearest neighbors
XGBoost: Extreme gradient boosting
LightGBM: Light gradient boosting machine
SHAP: Shapley Additive Explanations
CKD: Chronic kidney disease
