# Correction: Diagnostic efficacy of remnant cholesterol inflammatory index in diabetic kidney disease: machine learning approaches

**DOI:** 10.3389/fnut.2025.1755030

**Published:** 2025-12-15

**Authors:** Xili Xie, Xueyu Li, Haifeng Li, Yan Gao, Feng Zhao, Chen Jia

**Affiliations:** Department of Disease Prevention and Control, General Hospital of Northern Theater Command, Shenyang, Liaoning, China

**Keywords:** diabetic kidney disease, remnant cholesterol, inflammatory, machine learning, diagnostic model

The order of authors in the **Author List** of the published paper was erroneously given as:

Xili Xie, Haifeng Li, Yan Gao, Feng Zhao, Xueyu Li and Chen Jia^*^. The correct author list should read as: Xili Xie^†^, Xueyu Li^†^, Haifeng Li, Yan Gao, Feng Zhao and Chen Jia^*^.

Author Haifeng Li, Yan Gao, Feng Zhao was erroneously assigned as equal contribution. Author Xueyu Li was erroneously omitted as equal contributing author. The following authors provided equal contribution: Xili Xie and Xueyu Li.

The original version of this article has been updated.

