# Supplementary material for: Diagnostic efficacy of remnant cholesterol inflammatory index in diabetic kidney disease: machine learning approaches
Source: Front Nutr. 2025 Nov 25;12:1642358. doi: 10.3389/fnut.2025.1642358 (PMC12685678; doi:10.3389/fnut.2025.1642358)

Table S1. Characteristics of study participants before imputation

| Characteristics | Before Imputation |
| --- | --- |
| Age, years, mean ± SD | 50.77 ± 17.30 |
| Gender, N (%) |  |
| Male | 2889 (48.61) |
| Female | 3054 (51.39) |
| Race, N (%) |  |
| Mexican American | 845 (14.22) |
| Other Hispanic | 702 (11.81) |
| Non-Hispanic White | 2011 (33.84) |
| Non-Hispanic Black | 1383 (23.27) |
| Other | 1002 (16.86) |
| Educational level, N (%) |  |
| ≤High school diploma | 2617 (44.04) |
| >High school diploma | 2422 (55.96) |
| Marital status, N (%) |  |
| Married | 3350 (56.42) |
| Other | 2588 (43.58) |
| PIR, N (%) |  |
| <1 | 1053 (20.20) |
| ≥1 | 4161 (79.80) |
| Smoking status, N (%) |  |
| Yes | 2570 (43.30) |
| No | 3366 (56.70) |
| Drinking status, N (%) |  |
| Yes | 3897 (73.96) |
| No | 1372 (26.04) |
| Hypertension, N (%) |  |
| Yes | 2459 (45.21) |
| No | 2980 (54.79) |
| Diabetes duration, years, mean ± SD | 12.63 ± 10.89 |
| BMI, kg/m^2^, mean ± SD | 29.80 ± 7.39 |

Data are presented as number (%), mean ± standard deviation (SD), or median (interquartile range). Abbreviations: DKD, Diabetic Kidney Disease; BMI, body mass index.

Table S2. Associations of RC and RCII with glycated hemoglobin

| **Characteristics** | ***β* (95%CI)** |
| --- | --- |
| RC |  |
| Quartile 1 | 1.0 (Reference) |
| Quartile 2 | **0.11 (0.04, 0.19)** |
| Quartile 3 | **0.25 (0.16, 0.33)** |
| Quartile 4 | **0.50 (0.42, 0.59)** |
| *P* for trend | **<0.001** |
| Per-SD | **0.19 (0.16, 0.22)** |
| RCII |  |
| Quartile 1 | 1.0 (Reference) |
| Quartile 2 | **0.11 (0.02, 0.19)** |
| Quartile 3 | **0.27 (0.18, 0.36)** |
| Quartile 4 | **0.60 (0.50, 0.69)** |
| *P* for trend | **<0.001** |
| Per-SD | **0.14 (0.12, 0.18)** |

Adjusted for age, gender (male; female), race (Mexican American; Other Hispanic; Non-Hispanic Black; Non-Hispanic White; Other), educational level, (≤High school diploma; >High school diploma) marital status (Married; Other), PIR (<1; ≥1), smoking status (Yes; No), drinking status (Yes; No), BMI, Hypertension (Yes; No), and Diabetes duration.

Table S3. Associations of RC and RCII with albumin to creatinine ratio

| **Characteristics** | ***β* (95%CI)** |
| --- | --- |
| RC |  |
| Quartile 1 | 1.0 (Reference) |
| Quartile 2 | 26.53 (1.07, 52.00) |
| Quartile 3 | **36.50 (10.28, 62.71)** |
| Quartile 4 | **63.91 (36.77, 91.04)** |
| *P* for trend | **<0.001** |
| Per-SD | **24.28 (14.85, 33.71)** |
| RCII |  |
| Quartile 1 | 1.0 (Reference) |
| Quartile 2 | 16.33 (-9.93, 42.60) |
| Quartile 3 | **60.93 (32.94, 88.91)** |
| Quartile 4 | **95.45 (65.25, 125.66)** |
| *P* for trend | **<0.001** |
| Per-SD | **19.86 (10.38, 29.33)** |

Adjusted for age, gender (male; female), race (Mexican American; Other Hispanic; Non-Hispanic Black; Non-Hispanic White; Other), educational level, (≤High school diploma; >High school diploma) marital status (Married; Other), PIR (<1; ≥1), smoking status (Yes; No), drinking status (Yes; No), BMI, Hypertension (Yes; No), and Diabetes duration.

Table S4. Associations of RC and RCII with estimated glomerular filtration rate

| **Characteristics** | ***β* (95%CI)** |
| --- | --- |
| RC |  |
| Quartile 1 | 1.0 (Reference) |
| Quartile 2 | **-0.59 (-1.14, -0.03)** |
| Quartile 3 | **-1.88 (-2.45, -1.30)** |
| Quartile 4 | **-2.47 (-3.07, -1.87)** |
| *P* for trend | **<0.001** |
| Per-SD | **-0.87 (-1.08, -0.66)** |
| RCII |  |
| Quartile 1 | 1.0 (Reference) |
| Quartile 2 | -0.54 (-1.12, 0.05) |
| Quartile 3 | **-0.88 (-1.50, -0.26)** |
| Quartile 4 | **-0.71 (-1.38, -0.04)** |
| *P* for trend | **0.028** |
| Per-SD | -0.16 (-0.37, 0.05) |

Adjusted for age, gender (male; female), race (Mexican American; Other Hispanic; Non-Hispanic Black; Non-Hispanic White; Other), educational level, (≤High school diploma; >High school diploma) marital status (Married; Other), PIR (<1; ≥1), smoking status (Yes; No), drinking status (Yes; No), BMI, Hypertension (Yes; No) , and Diabetes duration.

Table S5. Associations of the component of RCII with the risk of Diabetic Kidney Disease

| **Characteristics** | **Model 1** | **Model 2** | **Model 3** |
| --- | --- | --- | --- |
|  | **OR (95%CI)** | **OR (95%CI)** | **OR (95%CI)** |
| hs-CRP |  |  |  |
| Quartile 1 | 1.0 (Reference) | 1.0 (Reference) | 1.0 (Reference) |
| Quartile 2 | 1.24 (1.00, 1.54) | 1.03 (0.82, 1.29) | 0.87 (0.68, 1.11) |
| Quartile 3 | **1.72 (1.40, 2.11)** | **1.52 (1.22, 1.89)** | 1.18 (0.93, 1.50) |
| Quartile 4 | **2.43 (1.99, 2.96)** | **2.51 (2.03, 3.11)** | **1.68 (1.31, 2.15)** |
| *P* for trend | **<0.001** | **<0.001** | **<0.001** |
| Per-SD | **1.23 (1.16, 1.31)** | **1.26 (1.18, 1.35)** | **1.16 (1.08, 1.24)** |
| TC |  |  |  |
| Quartile 1 | 1.0 (Reference) | 1.0 (Reference) | 1.0 (Reference) |
| Quartile 2 | **0.59 (0.50, 0.71)** | **0.61 (0.50, 0.74)** | **0.65 (0.53, 0.79)** |
| Quartile 3 | **0.41 (0.34, 0.50)** | **0.42 (0.34, 0.51)** | **0.46 (0.37, 0.57)** |
| Quartile 4 | **0.42 (0.35, 0.51)** | **0.38 (0.31, 0.47)** | **0.41 (0.33, 0.51)** |
| *P* for trend | **<0.001** | **<0.001** | **<0.001** |
| Per-SD | **0.70 (0.65, 0.75)** | **0.69 (0.64, 0.75)** | **0.72 (0.67, 0.78)** |
| HDL-C |  |  |  |
| Quartile 1 | 1.0 (Reference) | 1.0 (Reference) | 1.0 (Reference) |
| Quartile 2 | **0.66 (0.56, 0.79)** | **0.58 (0.48, 0.70)** | **0.59 (0.49, 0.72)** |
| Quartile 3 | **0.40 (0.33, 0.48)** | **0.31 (0.25, 0.38)** | **0.36 (0.28, 0.45)** |
| Quartile 4 | **0.27 (0.22, 0.34)** | **0.17 (0.14, 0.22)** | **0.24 (0.19, 0.32)** |
| *P* for trend | **<0.001** | **<0.001** | **<0.001** |
| Per-SD | **0.58 (0.53, 0.63)** | **0.48 (0.43, 0.53)** | **0.55 (0.50, 0.61)** |
| LDL-C |  |  |  |
| Quartile 1 | 1.0 (Reference) | 1.0 (Reference) | 1.0 (Reference) |
| Quartile 2 | **0.50 (0.42, 0.61)** | **0.57 (0.47, 0.69)** | **0.58 (0.47, 0.71)** |
| Quartile 3 | **0.39 (0.32, 0.47)** | **0.44 (0.36, 0.54)** | **0.42 (0.34, 0.52)** |
| Quartile 4 | **0.39 (0.32, 0.47)** | **0.42 (0.34, 0.51)** | **0.42 (0.34, 0.53)** |
| *P* for trend | **<0.001** | **<0.001** | **<0.001** |
| Per-SD | **0.69 (0.64, 0.74)** | **0.73 (0.67, 0.79)** | **0.74 (0.68, 0.80)** |

Model 1: Adjusted for none.

Model 2: Adjusted for age, gender (male; female).

Model 3: Adjusted for age, gender (male; female), race (Mexican American; Other Hispanic; Non-Hispanic Black; Non-Hispanic White; Other), educational level, (≤High school diploma; >High school diploma) marital status (Married; Other), PIR (<1; ≥1), smoking status (Yes; No), drinking status (Yes; No), BMI, Hypertension (Yes; No), and Diabetes duration.

Table S6. Sensitivity analysis for associations of RC and RCII with the risk of Diabetic Kidney Disease after further adjusting lipid-lowering drugs and antidiabetic drugs

| Characteristics | OR (95%CI) |
| --- | --- |
| RC |  |
| Quartile 1 | 1.0 (Reference) |
| Quartile 2 | 1.45 (0.95, 2.20) |
| Quartile 3 | **1.89 (1.26, 2.83)** |
| Quartile 4 | **2.32 (1.55, 3.50)** |
| *P* for trend | **<0.001** |
| Per-SD | **1.30 (1.15, 1.48）** |
| RCII |  |
| Quartile 1 | 1.0 (Reference) |
| Quartile 2 | 1.03 (0.67, 1.59) |
| Quartile 3 | **1.49 (0.98, 2.27)** |
| Quartile 4 | **2.69 (1.73, 4.18)** |
| *P* for trend | **<0.001** |
| Per-SD | **1.55 (1.33, 1.81)** |

Adjusted for age, gender (male; female), race (Mexican American; Other Hispanic; Non-Hispanic Black; Non-Hispanic White; Other), educational level, (≤High school diploma; >High school diploma) marital status (Married; Other), PIR (<1; ≥1), smoking status (Yes; No), drinking status (Yes; No), BMI, Hypertension (Yes; No), Diabetes duration, lipid-lowering drugs (Yes; No) and antidiabetic drugs (Yes; No).

Table S7. Sensitivity analysis for associations of RC and RCII with the risk of Diabetic Kidney Disease in Multiply Imputed Datasets

| **Characteristics** | **Model 1** | **Model 2** | **Model 3** |
| --- | --- | --- | --- |
|  | **OR (95%CI)** | **OR (95%CI)** | **OR (95%CI)** |
| RC |  |  |  |
| Quartile 1 | 1.0 (Reference) | 1.0 (Reference) | 1.0 (Reference) |
| Quartile 2 | **1.93 (1.52, 2.43)** | **1.58 (1.24, 2.02)** | **1.37 (1.07, 1.77)** |
| Quartile 3 | **3.13 (2.51, 3.91)** | **2.48 (1.96, 3.13)** | **1.99 (1.56, 2.54)** |
| Quartile 4 | **4.14 (3.32, 5.15)** | **3.49 (2.77, 4.39)** | **2.65 (2.08, 3.37)** |
| *P* for trend | **<0.001** | **<0.001** | **<0.001** |
| Per-SD | **1.47 (1.39, 1.56)** | **1.47 (1.38, 1.57)** | **1.37 (1.27, 1.47)** |
| RCII |  |  |  |
| Quartile 1 | 1.0 (Reference) | 1.0 (Reference) | 1.0 (Reference) |
| Quartile 2 | **1.50 (1.19, 1.89)** | 1.23 (0.97, 1.57) | 0.99 (0.77,1.27) |
| Quartile 3 | **2.28 (1.83, 2.83)** | **1.94 (1.54, 2.44)** | **1.37 (1.07, 1.75)** |
| Quartile 4 | **3.53 (2.86, 4.35)** | **3.47 (2.78, 4.33)** | **1.98 (1.54, 2.54)** |
| *P* for trend | **<0.001** | **<0.001** | **<0.001** |
| Per-SD | **1.42 (1.32, 1.52)** | **1.48 (1.37, 1.60)** | **1.28 (1.19, 1.38)** |

Model 1: Adjusted for none.

Model 2: Adjusted for age, gender (male; female).

Model 3: Adjusted for age, gender (male; female), race (Mexican American; Other Hispanic; Non-Hispanic Black; Non-Hispanic White; Other), educational level, (≤High school diploma; >High school diploma) marital status (Married; Other), PIR (<1; ≥1), smoking status (Yes; No), drinking status (Yes; No), BMI, Hypertension (Yes; No), and Diabetes duration.

Table S8. Sensitivity analysis for associations of RC and RCII with the risk of Diabetic Kidney Disease among 11271 participants in CHARLS cohort

| **Characteristics** | **Model 1** | **Model 2** | **Model 3** |
| --- | --- | --- | --- |
|  | **OR (95%CI)** | **OR (95%CI)** | **OR (95%CI)** |
| RC |  |  |  |
| Quartile 1 | 1.0 (Reference) | 1.0 (Reference) | 1.0 (Reference) |
| Quartile 2 | 0.99 (0.85, 1.14) | 0.99 (0.86, 1.15) | 0.95 (0.82, 1.11) |
| Quartile 3 | **1.19 (1.03, 1.37)** | **1.21 (1.05, 1.39)** | 1.13 (0.98, 1.31) |
| Quartile 4 | **1.42 (1.23, 1.63)** | **1.45 (1.26, 1.67)** | **1.28 (1.11, 1.47)** |
| *P* for trend | **<0.001** | **<0.001** | **<0.001** |
| Per-SD | **1.19 (1.14, 1.24)** | **1.20 (1.15, 1.25)** | **1.16 (1.11, 1.22)** |
| RCII |  |  |  |
| Quartile 1 | 1.0 (Reference) | 1.0 (Reference) | 1.0 (Reference) |
| Quartile 2 | 1.04 (0.90, 1.21) | 1.03 (0.89, 1.20) | 0.99 (0.85, 1.15) |
| Quartile 3 | **1.29 (1.12, 1.49)** | **1.26 (1.09, 1.45)** | 1.13 (0.98, 1.31) |
| Quartile 4 | **1.69 (1.47, 1.94)** | **1.64 (1.43, 1.88)** | **1.42 (1.23, 1.64)** |
| *P* for trend | **<0.001** | **<0.001** | **<0.001** |
| Per-SD | **1.22 (1.12, 1.33)** | **1.19 (1.10, 1.30)** | **1.14 (1.05, 1.24)** |

Model 1: Adjusted for none.

Model 2: Adjusted for age, gender (male; female).

Model 3: Adjusted for age, gender (male; female), educational level, (≤High school diploma; >High school diploma) marital status (Married; Other), household income (Low, High), smoking status (Yes; No), drinking status (Yes; No), BMI, Hypertension (Yes; No), and Diabetes duration.

Table S9. Multivariate logistic regression of the association between traditional risk factors and Diabetic Kidney Disease

| Traditional risk factors | *β* | OR (95%CI) | *P* |
| --- | --- | --- | --- |
| Age | **0.043** | **1.044 (1.037, 1.050)** | **<0.001** |
| Gender | **-0.500** | **0.607 (0.516, 0.713)** | **<0.001** |
| Race | **-0.097** | **0.907 (0.851, 0.968)** | **0.003** |
| Educational level | **-0.229** | **0.795 (0.679, 0.931)** | **0.004** |
| Marital status | **-0.283** | **0.754 (0.643, 0.883)** | **<0.001** |
| PIR | -0.102 | 0.903 (0.739, 1.103) | 0.318 |
| Smoking status | 0.056 | 1.058 (0.905, 1.236) | 0.480 |
| Drinking status | **0.423** | **1.526 (1.293, 1.800)** | **<0.001** |
| Hypertension | **0.878** | **2.406 (2.017, 2.869)** | **<0.001** |
| Diabetes duration | **0.025** | **1.025 (1.012, 1.038)** | **<0.001** |
| BMI, kg/m^2^ | **0.064** | **1.067 (1.053, 1.080)** | **<0.001** |

Table S10. Characteristics of study participants in the training set and testing set

|  | Training set | Testing set | *P* |
| --- | --- | --- | --- |
|  | N=4161 | N=1782 |  |
| Age, years, mean ± SD | 50.84 (17.22) | 50.60 (17.49) | 0.622 |
| Gender |  |  | 0.922 |
| Male | 2021 (48.57) | 868 (48.71) |  |
| Female | 2140 (51.43) | 914 (51.29) |  |
| Race |  |  | 0.730 |
| Mexican American | 594 (14.28) | 251 (14.09) |  |
| Other Hispanic | 484 (11.63) | 218 (12.23) |  |
| Non-Hispanic White | 1428 (34.32) | 583 (32.72) |  |
| Non-Hispanic Black | 965 (23.19) | 418 (23.46) |  |
| Other | 690 (16.58) | 312 (17.51) |  |
| Educational level |  |  | 0.138 |
| ≤High school diploma | 1807 (43.43) | 811 (45.51) |  |
| > High school diploma | 717 (56.57) | 1774 (54.49) |  |
| Marital status |  |  | **0.008** |
| Married | 2392 (57.49) | 958 (53.76) |  |
| Other | 1769 (42.51) | 824 (46.24) |  |
| Drinking status, N (%) |  |  | 0.433 |
| Yes | 3150 (75.70) | 1332 (74.75) |  |
| No | 1011 (24.30) | 450 (25.25) |  |
| Hypertension, N (%) |  |  | 0.570 |
| Yes | 1906 (45.81) | 802 (45.01) |  |
| No | 2255 (54.19) | 980 (54.99) |  |
| Diabetes duration, years, mean ± SD | 10.61 ± 5.41 | 10.76 ± 5.61 | 0.330 |
| BMI, kg/m^2^, mean ± SD | 29.79 ± 7.35 | 29.82 ± 7.47 | 0.891 |
| RCII, median (IQR) | 9.31 ± 18.96 | 9.13 ± 19.02 | 0.738 |

Data are presented as number (%), mean ± standard deviation (SD), or median (interquartile range). Abbreviations: BMI, body mass index; RCII, remnant cholesterol inflammatory index.

Table S11. The accuracy (95%CI) of seven machine learning models of RCII in the training set and testing set

| Model | Training set | Testing set |
| --- | --- | --- |
|  | Accuracy (95%CI) | Accuracy (95%CI) |
| LR | 0.840 (0.829, 0.851) | 0.830 (0.812, 0.847) |
| KNN | 0.988 (0.985, 0.991) | 0.854 (0.837, 0.870) |
| RF | 0.971 (0.965, 0.976) | 0.891 (0.876, 0.905) |
| XGBoost | 0.945 (0.938, 0.952) | 0.937 (0.924, 0.948) |
| LightGBM | 0.933 (0.925, 0.941) | 0.920 (0.906, 0.932) |
| SVM | 0.919 (0.911, 0.927) | 0.898 (0.884, 0.912) |

Abbreviations: LR: logistic regression; RF: random forest; SVM: support vector machine; KNN: K-nearest neighbors; XGB: extreme gradient boosting; LightGBM: Light Gradient Boosting Machine.

Table S12. Sensitivity analysis for the accuracy (95%CI) of seven machine learning models of RCII in the training set and testing set after Multiply Imputing

| Model | Training set | Testing set |
| --- | --- | --- |
|  | Accuracy (95%CI) | Accuracy (95%CI) |
| LR | 0.837 (0.826, 0.848) | 0.827 (0.809, 0.844) |
| KNN | 0.993 (0.990, 0.996) | 0.852 (0.835, 0.869) |
| RF | 0.963 (0.957, 0.968) | 0.888 (0.872, 0.902) |
| XGBoost | 0.902 (0.893, 0.911) | 0.893 (0.878, 0.907) |
| LightGBM | 0.917 (0.908, 0.925) | 0.900 (0.885, 0.913) |
| SVM | 0.907 (0.898, 0.916) | 0.892 (0.876, 0.906) |

Abbreviations: LR: logistic regression; RF: random forest; SVM: support vector machine; KNN: K-nearest neighbors; XGB: extreme gradient boosting; LightGBM: Light Gradient Boosting Machine.

Figure S1. Flowchart of Participant Selection


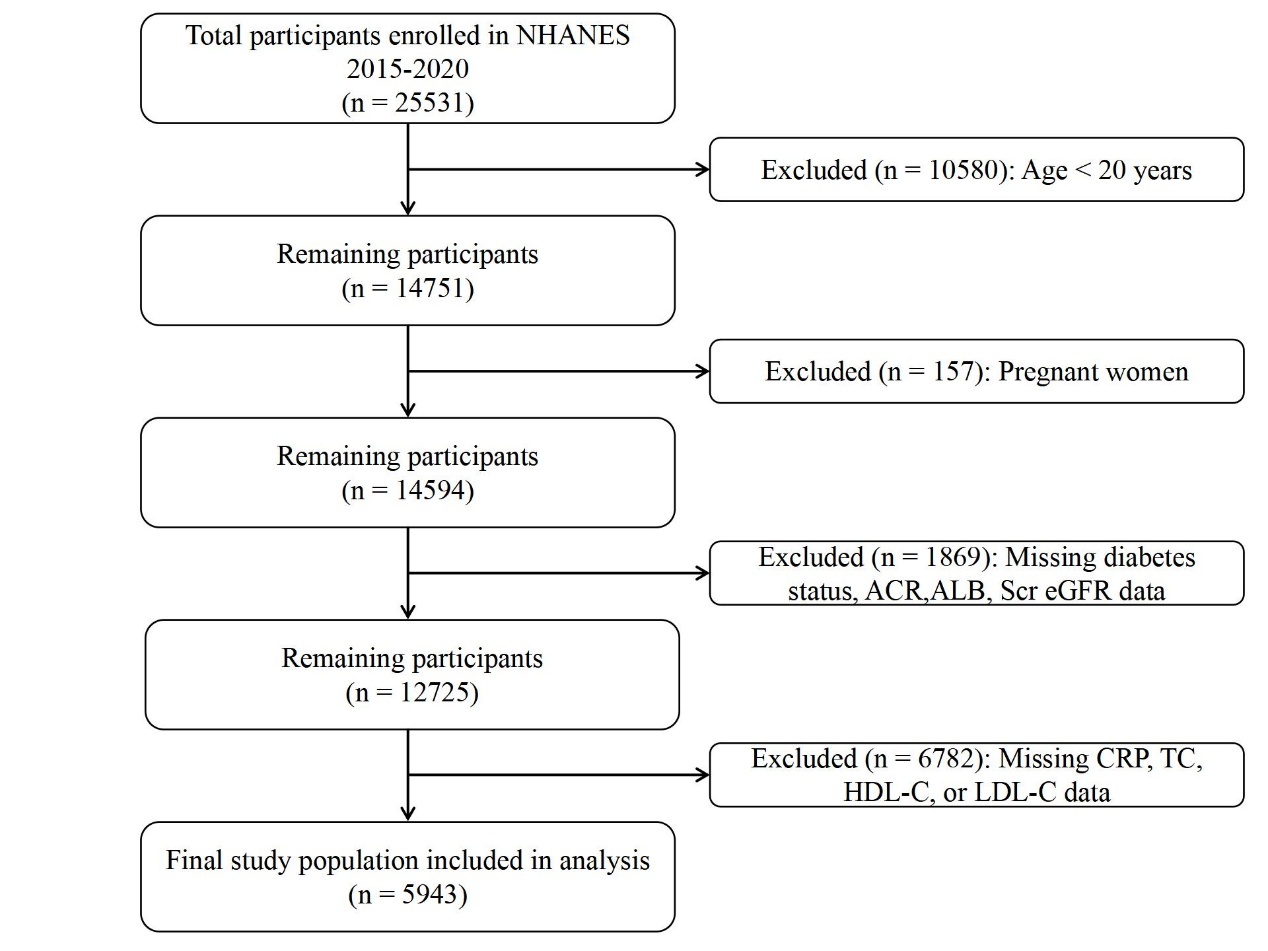


Figure S2. Sensitivity analysis for performance of machine learning (ML) models using RCII and traditional risk factors diagnosing Diabetic Kidney Disease after Multiply Imputing


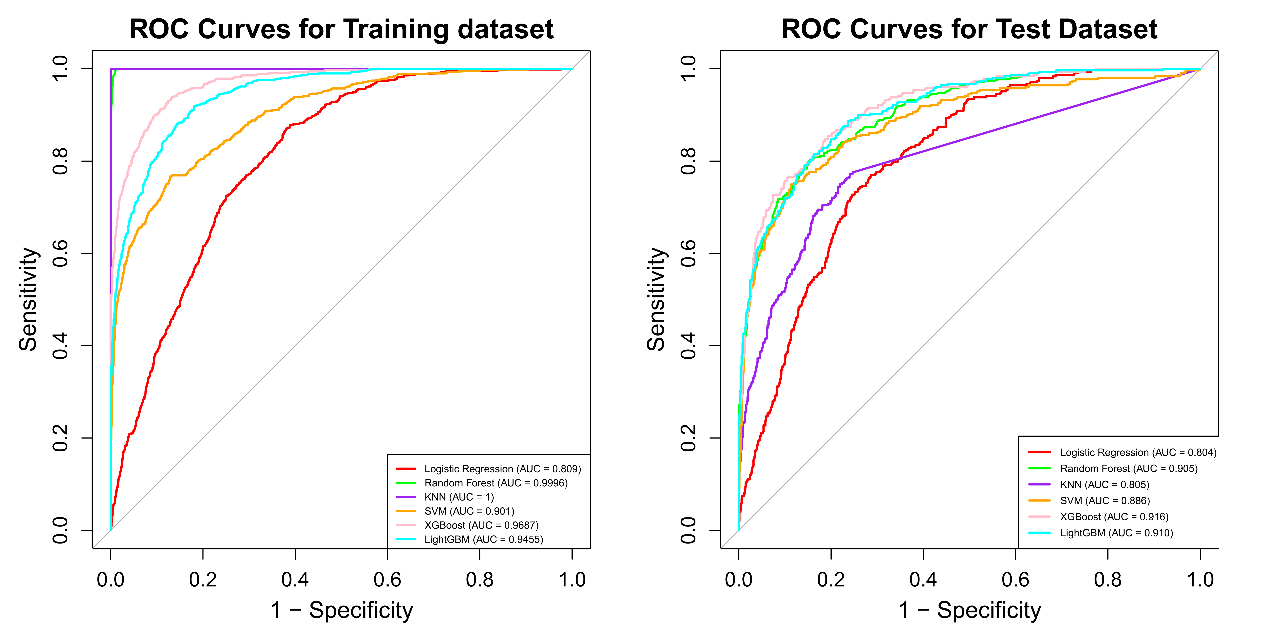


Figure S3. Sensitivity analysis for performance of XGBoost models using RCII and traditional risk factors diagnosing Diabetic Kidney Disease among 11271 participants in CHARLS cohort


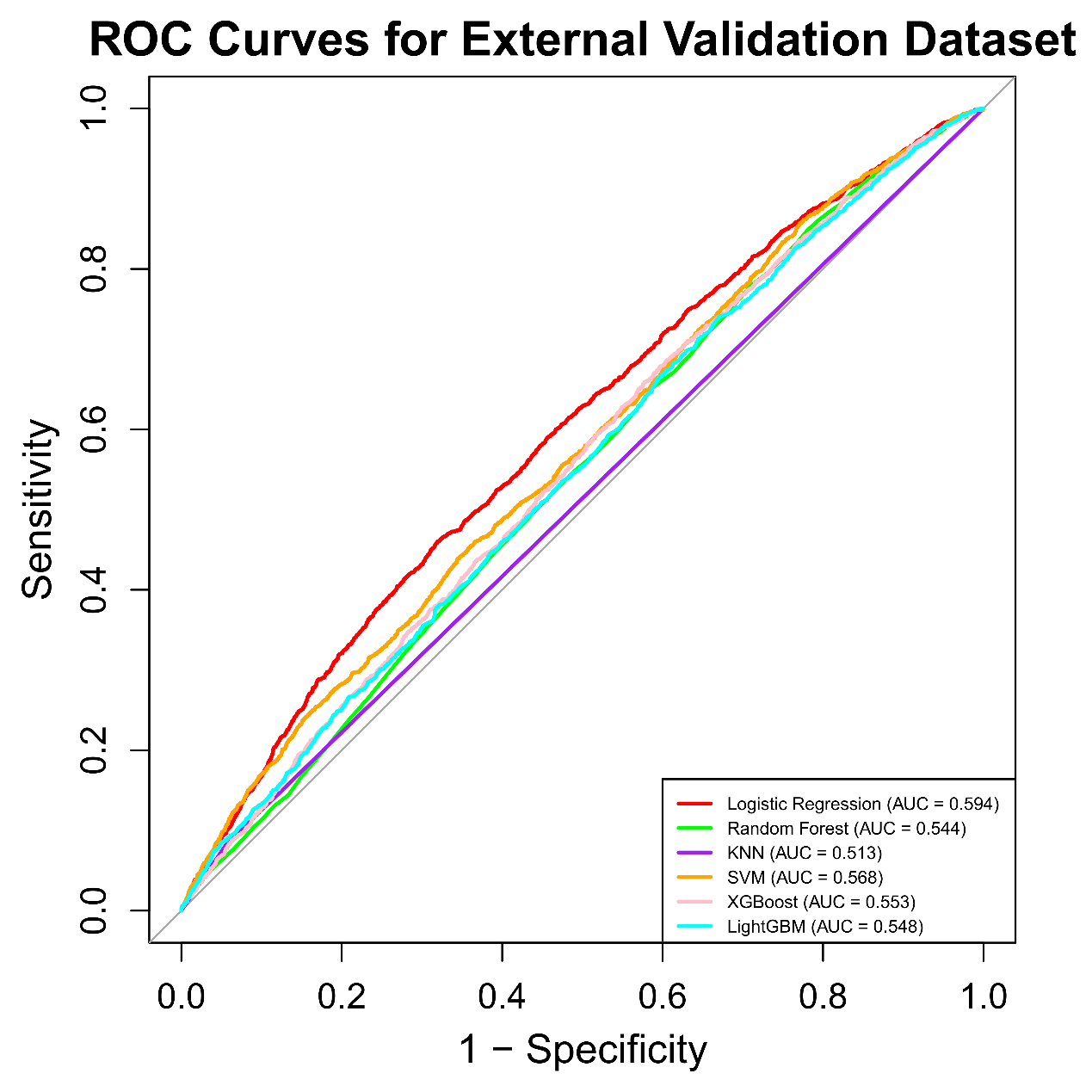

Supplement: Supplementary file 1 [file Data_Sheet_1.docx]
